# The Adenylyl Cyclase Activator Forskolin Increases Influenza Virus Propagation in MDCK Cells by Regulating ERK1/2 Activity

**DOI:** 10.4014/jmb.2306.06027

**Published:** 2023-09-04

**Authors:** Sang-Yeon Lee, Jisun Lee, Hye-Lim Park, Yong-Wook Park, Hun Kim, Jae-Hwan Nam

**Affiliations:** 1 Department of Medical and Biological Sciences, The Catholic University of Korea, Bucheon 14662, Republic of Korea; 2 Department of R&D, SK Bioscience, Seongnam 13493, Republic of Korea; 3 BK21 FOUR Department of Biotechnology, The Catholic University of Korea

**Keywords:** Influenza virus, forskolin, cAMP, ERKs, cell-based vaccine

## Abstract

Vaccination is the most effective method for preventing the spread of the influenza virus. Cell-based influenza vaccines have been developed to overcome the disadvantages of egg-based vaccines and their production efficiency has been previously discussed. In this study, we investigated whether treatment with forskolin (FSK), an adenylyl cyclase activator, affected the output of a cell-based influenza vaccine. We found that FSK increased the propagation of three influenza virus subtypes (A/H1N1/California/4/09, A/H3N2/Mississippi/1/85, and B/Shandong/7/97) in Madin–Darby canine kidney (MDCK) cells. Interestingly, FSK suppressed the growth of MDCK cells. This effect could be a result of protein kinase A (PKA)–Src axis activation, which downregulates extracellular signal-regulated kinase (ERK)1/2 activity and delays cell cycle progression from G_1_ to S. This delay in cell growth might benefit the binding and entry of the influenza virus in the early stages of viral replication. In contrast, FSK dramatically upregulated ERK1/2 activity via the cAMP–PKA–Raf-1 axis at a late stage of viral replication. Thus, increased ERK1/2 activity might contribute to increased viral ribonucleoprotein export and influenza virus propagation. The increase in viral titer induced by FSK could be explained by the action of cAMP in assisting the entry and binding of the influenza virus. Therefore, FSK addition to cell culture systems could help increase the production efficiency of cell-based vaccines against the influenza virus.

## Introduction

Influenza is a single-stranded RNA virus from the Orthomyxoviridae family that causes severe respiratory illnesses during seasonal or pandemic outbreaks [1, 2]. This virus has recently received considerable attention because of avian influenza outbreaks that occurred in Southeast Asia between 2003 and 2005, and the 2009 H1N1 pandemic [3]. Vaccination against influenza viruses is the most effective method for preventing viral infections and controlling viral spread [4]. Influenza vaccines are developed using egg-based systems. However, the manufacturing process faces several challenges, including a high risk of contamination during production and difficulty in meeting unexpected increases in demand [5]. Consequently, during the last flu pandemic (H1N1/09), the spread of the infection increased exponentially and supplying sufficient amounts of the vaccine was challenging [6]. To overcome this issue, cell-based vaccines (which are faster to produce, more flexible, and easier to control than egg-based vaccines) have been developed [4]. However, several problems remain unresolved, such as the high cost and low yield of egg-based vaccines [7]. Numerous attempts have been made to establish a system for producing high-yield influenza vaccines, including the development of novel modified cell lines to improve influenza virus [8-10]. For example, Hamamoto *et al*. [7] demonstrated that Madin–Darby canine kidney (MDCK) cells with a stable knockdown of the gene encoding interferon regulatory factor 7 produced a high yield of influenza virus. However, the Food and Drug Administration must reapprove each of these types of vaccine before commercial production can commence, and vaccines have limited applications in several production processes.

Adenylyl cyclase (AC) is an enzyme with an essential role in all cells. AC catalyzes the conversion of adenosine triphosphate to 3',5'-cyclic adenosine monophosphate (cAMP) [11], which is involved in a wide range of cellular processes (including cell proliferation, apoptosis, immune response, and neurohormonal signaling) as a second messenger [12, 13]. Intracellular cAMP levels can be elevated by various agents including forskolin (FSK), prostaglandin E2 (PGE2), and 3-isobutyl-1-methylxanthine (IBMX) [14, 15].

For instance, PGE2 is a lipid molecule that acts as a signaling molecule to trigger the production of cAMP through G protein-coupled receptors in the body. Conversely, IBMX extends the duration of cAMP signaling by indiscriminately impeding PDE (Cyclic nucleotide phosphodiesterases) activity [16]. Among these agents, FSK, a natural compound, is a direct activator of adenylate cyclase, distinguishing it from PGE2 and IBMX [17]. Thus, the focus of this investigation revolved around the functions of FSK and its underlying mechanisms in the context of generating influenza vaccines with enhanced yields. FSK was used to study the role of cAMP as a direct activator of AC [18]. FSK are also widely used in healthcare products. cAMP regulates the activation or inhibition of cell growth depending on cell type [19]. For example, FSK and heregulin synergistically induce Schwann cells [20]. However, FSK increases intracellular cAMP levels and inhibits the proliferation of NIH3T3 cells [21]. In addition, FSK induces macrophage proliferation and inhibits T cell proliferation via the cAMP pathway [22, 23]. cAMP regulates cell proliferation through cAMP-dependent protein kinase A (PKA) and mitogen-activated protein kinase (MAPK) cascades, and extracellular signal-regulated kinase (ERK)1/2 [19]. The ability of cAMP to inhibit cell proliferation relies on its action as an inhibitor of Raf-1 activation via Src and Rap1, which consequently blocks ERK1/2 activity [24, 25]. In addition, cAMP mediates cell cycle suppression by increasing p21 levels and decreasing cyclin D1 levels [26, 27]. During viral replication, the host cell cycle is important for viral spread, and specific viruses prefer specific phases of the cell cycle [28, 29]. The influenza virus takes advantage of the G_0_/G_1_ phase of host cells because of the higher sialic acid content and lower lipid content in G_1_ than in the S/G_2_/M phase [30, 31].

This study investigated the potential of FSK, an activator of AC, in enhancing influenza virus propagation. The findings showed that the titer of the influenza virus increased by 120–300% in FSK-supplemented culture medium. Furthermore, in MDCK cells stimulated with FSK, cell proliferation was inhibited and the cell cycle was delayed at the G_1_ phase at an early stage of infection. In contrast, ERK1/2 activity increased at later stages of viral infection. We suggest that coordinated regulation of ERK1/2 activity contributes to influenza virus propagation by supporting viral entry and budding.

## Materials and Methods

### Cells, Viruses, and Infection

MDCK cells were maintained in Minimum Essential Medium (MEM; Gibco BRL, USA) supplemented with 10% (v/v) fetal bovine serum (FBS) and 1% (v/v) antibiotic-antimycotics. The cells were cultured at 37°C under 5% CO_2_ in humidified air. The influenza viruses (A/H1N1/California/04/09, A/H3N2/Mississippi/1/85, and B/Shandong/7/97) were provided by Dr. Baik-lin Seong (Yonsei University, Seoul, Korea) and were used to infect MDCK cells. The cells were washed with phosphate-buffered saline (PBS) and infected at a multiplicity of infection (MOI) in serum-free (SF) medium for 1 h at 37°C. After infection, the cells were incubated with SF MEM containing 1 μg/ml L-(Tosylamide-2-phenyl) ethyl chloromethyl ketone (TPCK)-treated trypsin (Sigma-Aldrich, USA) with or without 12.5 μM FSK (AG Scientific). FSK was dissolved in ethyl acetate as a 10 mg/ml stock solution. SQ22,536 (Sigma-Aldrich) was dissolved in sterile water to a stock concentration of 25 mM and used at a concentration of 20 μM. Cells and supernatants were collected at the indicated time points after infection to determine the yield of infectious virus particles.

### Viral Titration

The viral titer was determined using a plaque assay. Confluent monolayers of MDCK cells in 35 mm dishes (Corning, USA) were inoculated with influenza virus diluted serially 10-fold and incubated at 37°C for 1 h. The inoculum was removed and overlaid with Dulbecco’s modified Eagle’s medium (DMEM) containing 1% (w/v) agarose and 0.001% (v/v) trypsin. After incubation for 3 days at 37°C, the cells were fixed with methanol/acetic acid (3:1) and stained with crystal violet solution (Sigma-Aldrich). The number of plaques was evaluated and expressed as plaque-forming units (PFU/ml).

### Flow Cytometry

MDCK cells were infected with A/H1N1/4/09 virus. The infected cells were incubated for 24 and 48 h in SF medium containing 1 μg/ml TPCK-treated trypsin with or without FSK (12.5 μM), detached using trypsin-EDTA, and centrifuged at 1,500 rpm for 5 min. The cells were stained with a primary antibody (Ab), anti-influenza A-hemagglutinin (HA) (Thermo Scientific Pierce, USA). After two washes with a buffer containing 3% (v/v) FBS and 0.02% (v/v) sodium azide in PBS, the cells were stained with an anti-rabbit secondary antibody tagged with fluoscein isothiocyanate (Bethyl) and fixed with 1% (v/v) paraformaldehyde. Finally, cells expressing the HA of the influenza virus were analyzed using flow cytometry (Canto II; BD Bioscience, USA) and FlowJo software.

### Cell Proliferation Assays

For the cell counting kit-8 (CCK-8) assay, MDCK cells were pre-incubated for 24 h in 96-well plates (Corning) at a density of 1 × 10^5^ cells/well. After adding various concentrations of FSK, the cells were incubated for 6, 12, 24, and 48 h at 37°C. The CCK-8 solution (Dojindo, USA) was added to each well at the indicated time points. The color was developed for 2 h and the absorbance was measured at 450 nm using a microplate reader (Thermo Scientific Pierce, USA).

### Wound-Healing Assays

MDCK cells were cultured to near confluence (>90% confluence) in six-well plates (Corning,). The cells were rinsed with PBS twice and starved overnight (O/N) in SF medium. Using a sterile 200 μl pipet tip, the cell layers were scratched to form wounds in a grid pattern. The cells were rinsed with PBS, which was then replaced with fresh medium containing 12.5 μM or 100 μM FSK. The cells were incubated for 18 h and observed under an inverted microscope (Olympus Co., Japan).

### Cell Cycle Analysis

MDCK cells were seeded at 25–30% confluence in 60 mm dishes (Corning). For cell synchronization, 2 mM thymidine (Sigma-Aldrich) was added, followed by incubation for 19 h at 37°C. After washing with PBS, fresh medium was added. The cells were incubated for 9 h at 37°C and exposed to 2 mM thymidine O/N for a second thymidine block. After cell synchronization, the medium was replaced with fresh medium with or without 12.5 μM FSK. Cells were collected at various times (2, 6, 12, or 24 h) and fixed in 75% ethyl alcohol for at least 2 h at –20°C. After washing, the cells were stained with propidium iodide (PI) staining buffer (BD Biosciences) for 15 min at room temperature (RT) and analyzed using a flow cytometer (Accurri; BD Biosciences, USA) and FlowJo software.

### Western Blotting

MDCK cells were infected with or without influenza virus A/H1N1/4/09. After 1 h of inoculation, the infected cells were incubated with SF medium containing 1 μg/ml TPCK-treated trypsin with or without 12.5 μM FSK for 2, 6, or 24 h at 37°C. Non-infected MDCK cells were also incubated with complete medium with or without 12.5 μM FSK for the same time and conditions as the infected cells. The cells were collected at the indicated times and centrifuged at 4,000 rpm for 10 min. The pellets were lysed in RIRA buffer containing a protease inhibitor cocktail, PMSF (phenylmethylsulfonyl fluoride), and Na-orthovanadate (Santa Cruz Biotechnology, USA), incubated on ice for 10 min, and centrifuged at 13,000 rpm for 10 min. The protein concentration was determined using the Bradford assay. Proteins were separated using sodium dodecyl sulphate polyacrylamide gel electrophoresis and blotted onto polyvinylidene fluoride membranes (Bio-Rad, USA). The following primary antibodies were used: p-ERK1/2 (Santa Cruz Biotechnology), total-ERK1/2 (Cell Signaling Technology, USA), EPAC (Enzo Life Sciences, PA), Raf-1 (Santa Cruz Biotechnology), p-Src (Cell Signaling Technology), cyclin D1 (Cell Signaling Technology), p21 (Santa Cruz Biotechnology), cyclin-dependent kinase 4 (CDK4; Santa Cruz Biotechnology, USA), GAPDH (Ab Frontier), and a-tubulin (Ab Frontier, USA). Secondary antibodies were used to target each primary antibody for western blot analysis. The relative levels of the proteins were quantified using ImageJ from the NIH (USA).

### PKA Assay

Active PKA was determined using a PKA kinase activity kit (Abcam, UK) following the manufacturer’s instructions. Briefly, MDCK cells were treated with or without the influenza A/H1N1/4/09 virus. After 1 h inoculation, infected cells were incubated with SF medium containing 1 μg/ml TPCK-treated trypsin with or without 12.5 μM FSK for 2, 6, and 24 h at 37°C. Non-infected MDCK cells were also incubated with complete medium with or without 12.5 μM FSK using the same times and conditions as the infected cells. All cells were lysed using a protein lysis buffer containing a protease inhibitor cocktail (Thermo Scientific Pierce) and phenyl-methylsulfonyl fluoride (Santa Cruz Biotechnology) and harvested by centrifugation at 13,000 rpm for 15 min. The supernatants were tested using the Bradford assay to quantify protein content. The PKA substrate microtiter plate was activated using a kinase assay dilution buffer. Aliquots of 2 μg of cell lysates and ATP were loaded onto the plate and incubated for 1 h and 30 min at 30°C. After stopping the reaction by emptying the contents of each well, a phospho-specific substrate antibody was added to the plate, followed by incubation for 1 h at RT. The wells were washed four times with 1 × wash buffer. Anti-rabbit IgG-horseradish peroxidase conjugate was added at a 1:1000 dilution, and incubation was continued for 30 min at RT. After washing, tetramethylbenzidine substrate was added for color development, and the reaction was terminated with a stop solution. The absorbance was measured at 450 nm using a microplate reader (Thermo Scientific Pierce).

### Statistical Analysis

Statistical analyses were conducted using the Prism 5 software (GraphPad, USA). All data are expressed as mean± standard deviation (SD). Any significant differences between means were analyzed using Student’s *t* test. Differences at *p* < 0.05 were considered statistically significant.

## Results

### Forskolin (FSK) Treatment Increased the Titers of Three Influenza Viruses Used as Vaccine Strains in MDCK Cells

**Fig. 1:**
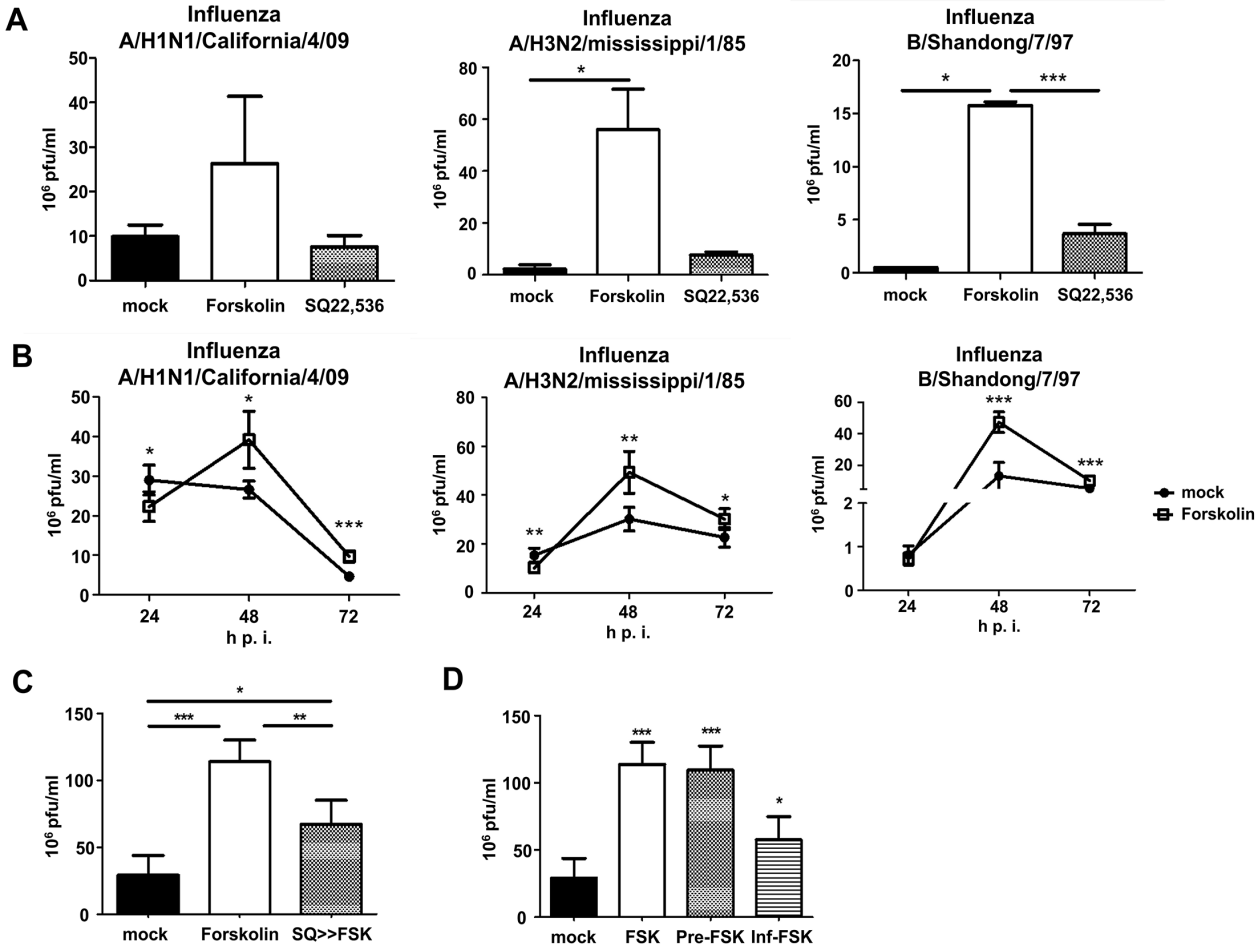
The effect of adenyl cyclase (AC) activator on the propagation of the influenza virus. Madin–Darby canine kidney (MDCK) cells were infected with the A/H1N1/California/04/09 virus at a multiplicity of infection (MOI) of 0.002 and treated with or without an AC activator or inhibitor. The culture supernatant was collected at 48 h p.i. and the infectious virus was determined using a plaque assay. (**A**) The effect of AC on influenza virus replication. (**B**) The rate of virus growth was determined over 3 days p.i. (**C**) The effect of an AC activator [forskolin (FSK)] on the action of the AC inhibitor SQ22,536. (**D**) The effects of FSK were dependent on the treatment time relative to viral infection: FSK, treatment after infection; pre-FSK, treatment before infection; inf-FSK, treatment with infection at the same time; mock, viral infection without FSK treatment. Results are shown as the mean ± SD (*n* = 4–6); *p < 0.05; **p < 0.01; and ***p < 0.001.

Previous reports have demonstrated the effects of AC on viral infections such as HIV-1 [32]. However, the underlying mechanism of the functional association between the influenza virus and AC is not well understood. We investigated influenza virus propagation under AC activation and inhibition. Three viral subtypes (A/H1N1/California/04/09, A/H3N2/Mississippi/1/85, and B/Shandong/7/97) that were components of recent influenza vaccines, were examined. FSK, a direct activator of AC, dramatically increased the titers of all three viral types. Conversely, cells treated with the AC inhibitors SQ22,536 showed no differences compared to the control group (mock) (Fig. 1A). These data demonstrate that FSK stimulates viral propagation through AC activation, whereas AC inhibitors do not affect viral propagation. To determine the yield of influenza virus in the presence of FSK, we established an influenza virus growth curve. Viral titers were determined using plaque assays 24, 48, and 72 h after infection. All three subtypes exhibited similar patterns over time. As shown in Fig. 1B, the viral titers in cells treated with FSK were significantly increased at 48 h compared to the viral titer at 24 h after infection. Comparing the viral titers in the cells treated with or without FSK (mock or FSK), the viral titers at 48 h after infection were higher than those of the mock, whereas the upregulated levels decreased to similar levels as the mock at 72 h after infection (Fig. 1B). Next, we investigated the effects of an AC inhibitor on FSK-induced viral propagation. Application of the inhibitor decreased the production of viruses compared to FSK treatment alone (Fig. 1C). These results indicated that FSK-induced AC activation played a critical role in influenza virus replication.

We investigated how the effect of FSK depended on the treatment sequence before, after, or simultaneously with influenza virus infection. FSK treatment before infection (pre-FSK) resulted in titers as high as those when FSK was applied after infection (FSK). Additionally, FSK treatment simultaneously with infection (inf-FSK) slightly increased the titers compared to the controls (no treatment with FSK; Fig. 1D). These findings indicated that the FSK treatment sequence for AC activation before and after viral infection had a beneficial effect on influenza virus replication.

Based on the finding that FSK treatment concurrently with viral infection did not affect viral propagation, we performed further studies to determine how the effect of FSK on influenza virus production depends on MDCK cell density. The viral titer increased to 60% confluence in the presence of FSK. At 30% and 100% confluence, FSK did not affect the increased output of influenza virus (Fig. S1). These data suggest that FSK differentially regulates certain cellular processes such as the cell cycle in a cell density-dependent manner.

### Forskolin (FSK) Treatment Inhibited the Proliferation of MDCK Cells

**Fig. 2:**
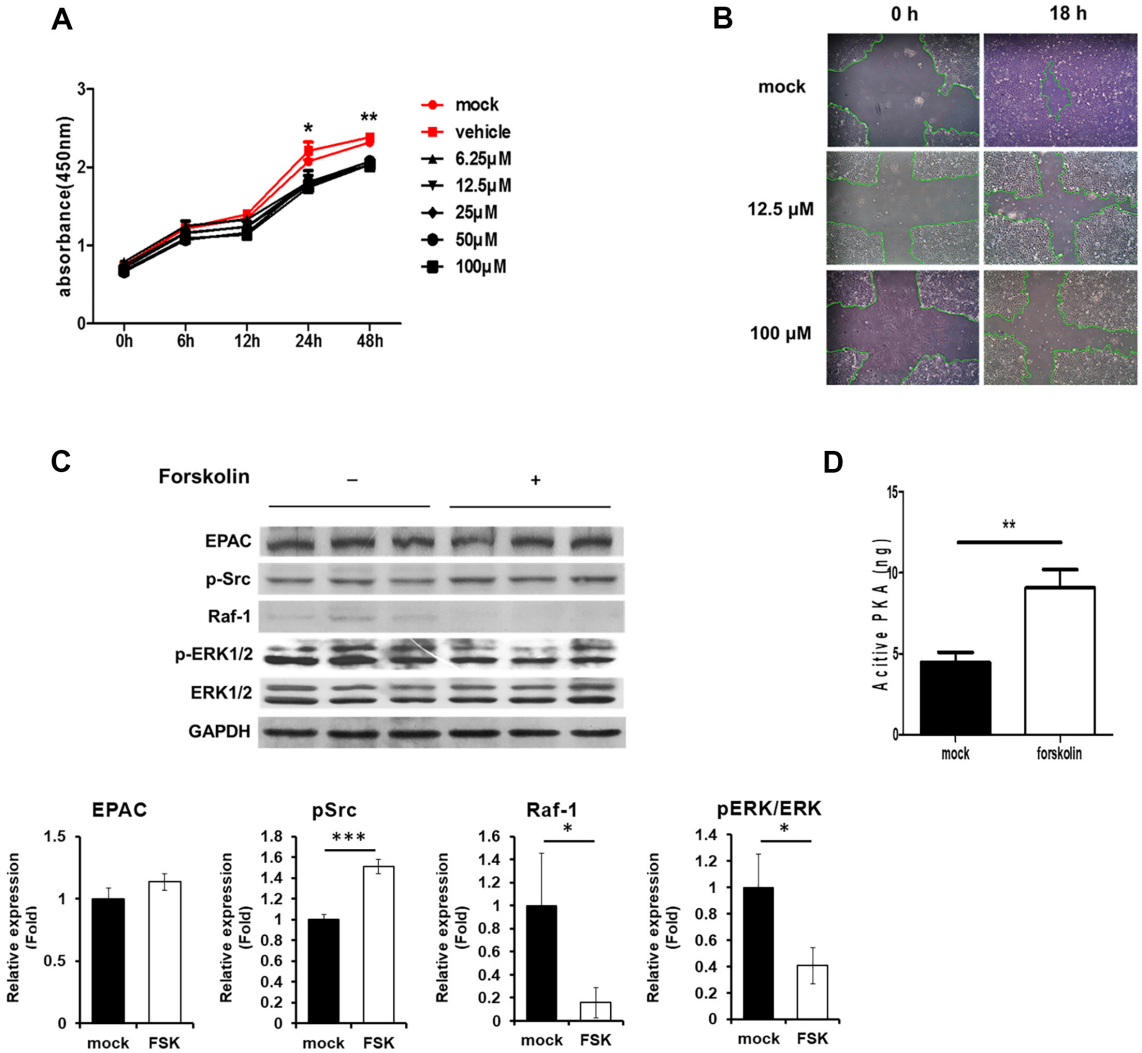
Inhibition of Madin–Darby canine kidney (MDCK) cell proliferation by forskolin (FSK). MDCK cells were incubated with various concentrations of FSK for the indicated times. (**A**) Cell counting kit-8 (CCK-8) assays were used to analyze MDCK cell proliferation. (**B**) Confluent MDCK cells were starved of serum overnight and the layer was scratched in a grid fashion. Cells were then incubated with or without FSK for 18 h. (**C**) Cells were incubated with (+) or without (–) 12.5 μM of FSK and were then harvested after 24 h of treatment for western blotting. The levels of EPAC, pSrc, and Raf-1 are expressed as the ratio of the densitometric measurement of the corresponding internal standard (GAPDH) according to densitometric analysis. The level of pERK is expressed as the ratio of the phosphorylated proteins to the corresponding total proteins according to densitometric analysis. (**D**) The activity of cAMP-dependent protein kinase A (PKA) was measured using assay kits in the same conditions as (**C**). Results are shown as the mean ± SD; *p < 0.05, **p < 0.01 and ***p < 0.001.

cAMP acts as an activator or inhibitor of cell proliferation, depending on the cell type [19]. We investigated whether cAMP activated or inhibited MDCK cell proliferation using a CCK-8 assay. The proliferation rate of the FSK-treated cells was lower than that of the control group (mock and vehicle; Fig. 2A). To investigate whether FSK affects cell migration, the cell layers were scratched using sterile microtips after starving the confluent MDCK cells. Cells were treated with FSK at different concentrations (12.5 μM and 100 μM) and observed at 18 h after treatment. Wound healing decreased in FSK-treated cells compared to that in mock cells, indicating that FSK inhibited not only cell proliferation but also cell migration (Fig. 2B). Based on these results, we investigated the association between cAMP levels and signaling pathways involved in cell growth. The ERK1/2 signaling pathway is a fundamental cellular cascade that orchestrates diverse cellular processes, including cell growth, differentiation, survival, and proliferation. This pathway is intricately regulated by multiple components, of which Raf-1, PKA, and EPAC play pivotal roles [33]. In this context, the signaling pathway was examined in cells 24 h after FSK treatment using western blotting. Notably, the phosphorylation of Src wasincreased and the expression of EPAC did not change. In contrast, both phosphorylation of ERK1/2 and expression of Raf-1 decreased in FSK-treated cells (Fig. 2C). In addition, PKA activity doubled in FSK-treated cells (Fig. 2D). These findings suggested that FSK treatment increased the Src phosphorylation by activating PKA, subsequently inhibiting Ras-dependent Raf-1 activation and resulting in diminished activity of ERK1/2 in FSK-treated cells.

### Forskolin Treatment Delayed the Cell Cycle Leading to Accumulation at the G_0_/G_1_ Phases in MDCK Cells

**Fig. 3:**
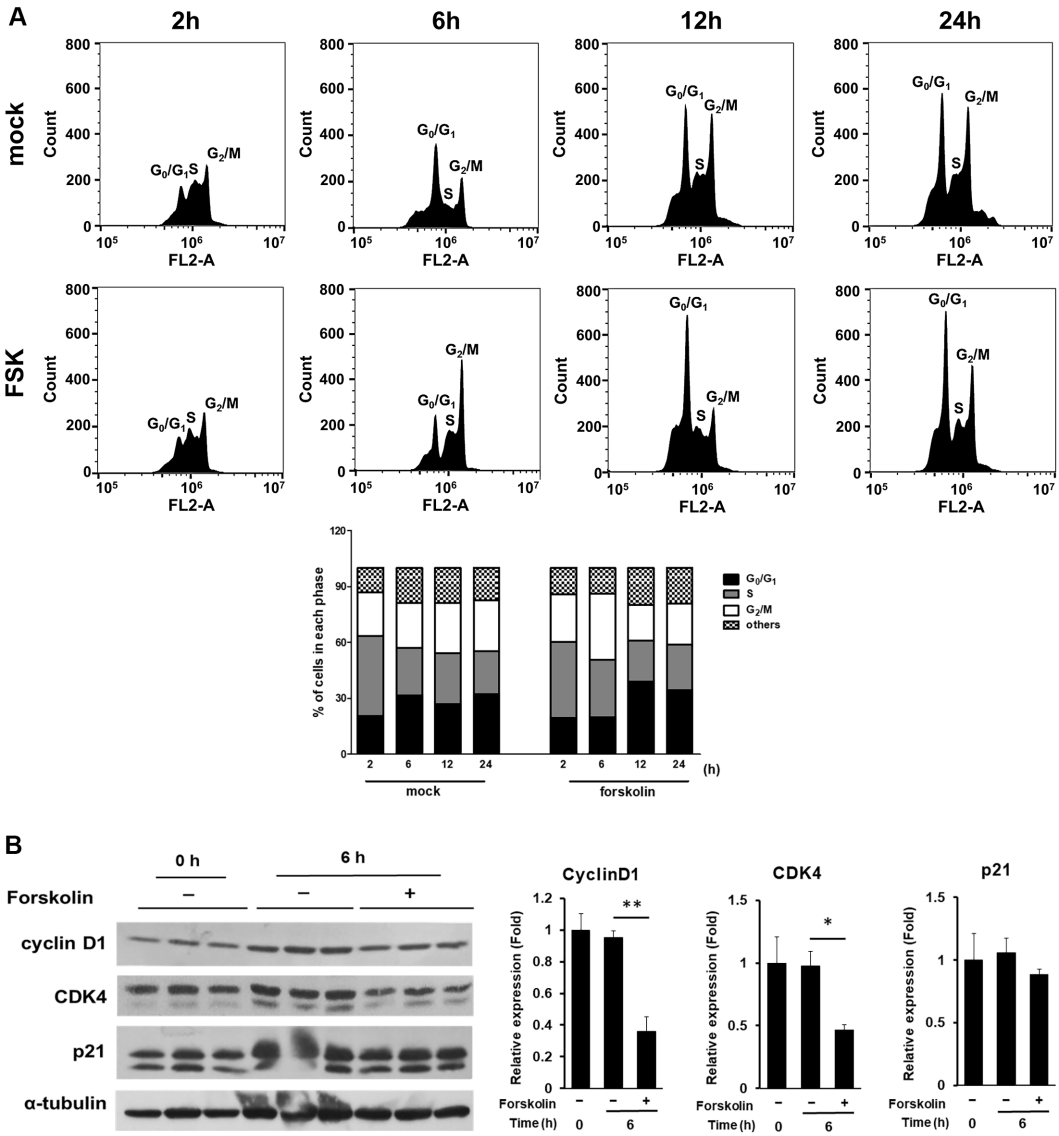
Analysis of cell cycle dynamics in the presence or absence of forskolin (FSK). (**A**) Madin–Darby canine kidney (MDCK) cells were incubated with or without FSK (mock) for the indicated times. The cells were then harvested for propidium iodide (PI) staining and analyzed using flow cytometry. The histogram presents the cell cycle phase distribution and the stacked bar graph shows mean data. The mean ± SD values are listed in Table S1. (**B**) Cell cycle regulatory molecules were analyzed using western blotting under the same conditions at 6 h after release from synchronization. The factors were proteins involved in the control of the G_0_/G_1_ to S phase transition: cyclin D1, p21, and CDK4. The levels of cyclin D1, p21, and CDK4 are expressed as the ratio of the densitometric measurement of the corresponding internal standard (α-tubulin) according to densitometric analysis. Data are shown as the mean (*n* = 3). Results are shown as the mean ± SD; *p < 0.05 and **p < 0.01.

FSK treatment inhibited MDCK cell proliferation (Fig. 2). Based on this finding, we investigated whether FSK was involved in cell cycle progression. MDCK cells were synchronized in the G_1_/S phase using double thymidine blockage. Subsequently, by adding complete medium with or without FSK, the cells were released into the S phase. As shown in Fig. 3A, 6 h after FSK treatment, the cell population in the S phase was initially much higher than that in the untreated control cells (mock). Cell cycle recovery of untreated control cells was faster than that of FSK-treated cells (Fig. 3A). This indicated that FSK delayed the cell cycle of MDCK cells at the time of cell cycle release. Twelve hours after treatment, cells in the FSK-treated group accumulated in the G_0_/G_1_ phase (Fig. 3A and Table S1). In addition, we examined the molecules involved in cell cycle regulation by focusing on G_0_/G_1_ to S phase transition. For western blotting, the MDCK cells were synchronized and treated with or without FSK. The cells were incubated for 6 h and analyzed using appropriate antibodies. The expression of cyclin D1 and CDK4 decreased in the FSK-treated cells, whereas the expression of p21 (a cell cycle inhibitor) did not differ between the mock- and FSK-treated groups (Fig. 3B). These findings indicated that obstruction of cell cycle progression was also involved in the inhibition of MDCK cell proliferation by FSK treatment.

### ERK1/2 Plays a Role in Late Events of the Influenza Virus Replication in the Presence of FSK

**Fig. 4:**
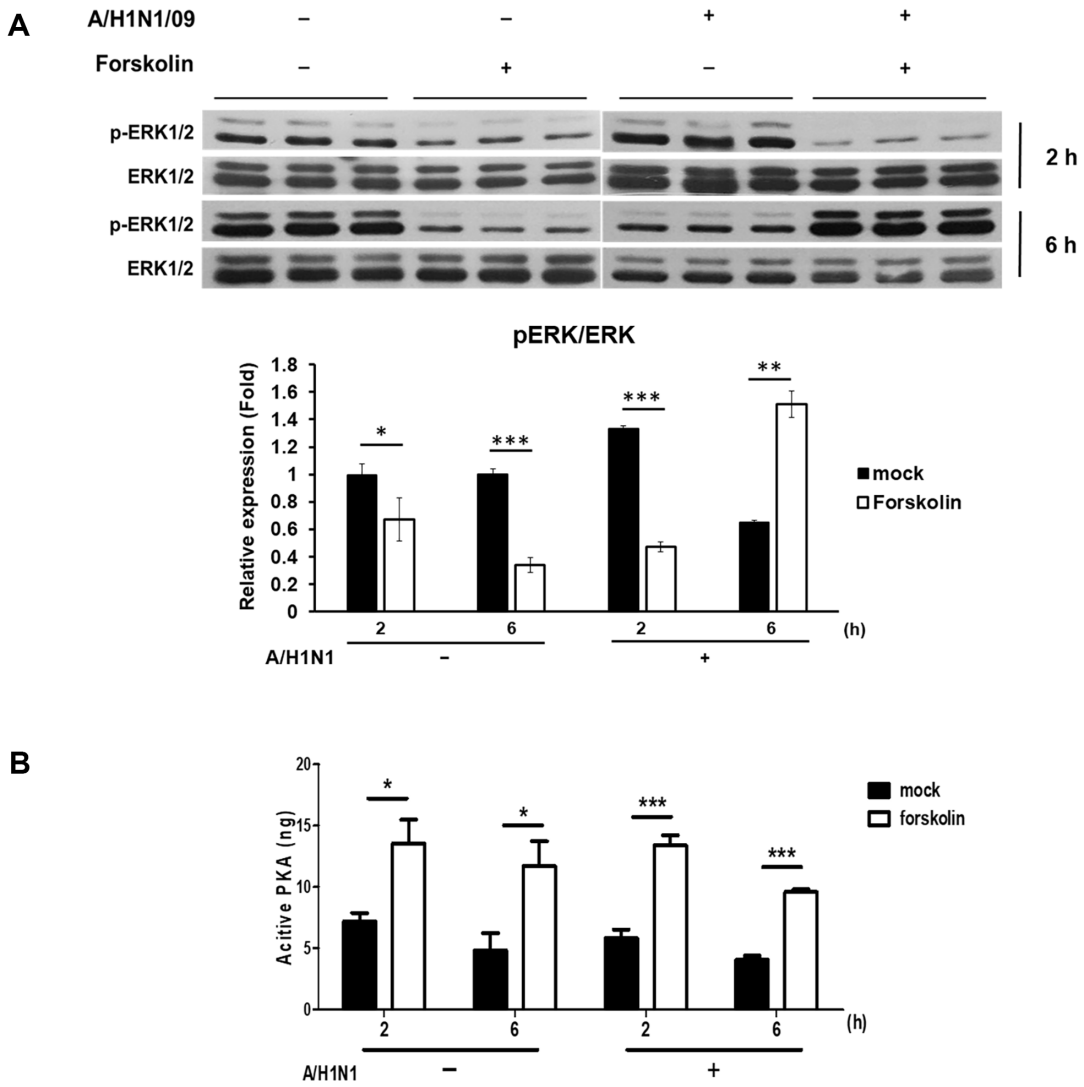
Activation of extracellular signal-regulated kinases (ERK)1/2 at a late phase of influenza virus replication. (**A**) MDCK cells were infected with or without 1 MOI of A/H1N1/California/04/09 virus in the presence or absence of FSK. The cells were harvested after 2 and 6 h for western blotting. The level of pERK is expressed as the ratio of the phosphorylated proteins to the corresponding total proteins according to densitometric analysis. (**B**) The activity of PKA was measured using PKA assay kits in the same conditions as (**A**). Mock indicates no treatment with FSK. Data are shown as the mean (*n* = 3); *p < 0.05, **p < 0.01 and ***p < 0.001.

ERKs (classical MAP kinases) are typically involved in cell proliferation, differentiation, immune responses, and anti-apoptosis [33]. Moreover, ERKs participate in influenza virus replication signaling [34]. Additionally, influenza viral infection is divided into two distinct phases: an early phase (lasting 2.5 h) and a late phase (lasting at least 5.5 h) [35, 36]. We verified the activity of ERK1/2 in cells treated with FSK and subjected them to influenza virus infection. MDCK cells were stimulated with FSK (12.5 μM) for 2 and 6 h after treatment with or without the influenza virus at an MOI of 1. Interestingly, 6 h after FSK treatment, ERK1/2 activation increased dramatically in FSK-treated infected cells but decreased in FSK-treated cells without infection. However, 2 h after FSK treatment, ERK1/2 activation decreased in FSK-treated cells regardless of their infection status (Fig. 4A). Nonetheless, no differences were observed in the activation of PKA, an upstream molecule of ERK1/2 upon infection (Fig. 4B). Thus, ERK1/2 activation increases during the late stages of influenza virus replication, independent of PKA activation, 6–8 h p.i.

### Forskolin Treatment Increased the Expression of Influenza Virus HA Protein, Which Increased ERK1/2 Activation

**Fig. 5:**
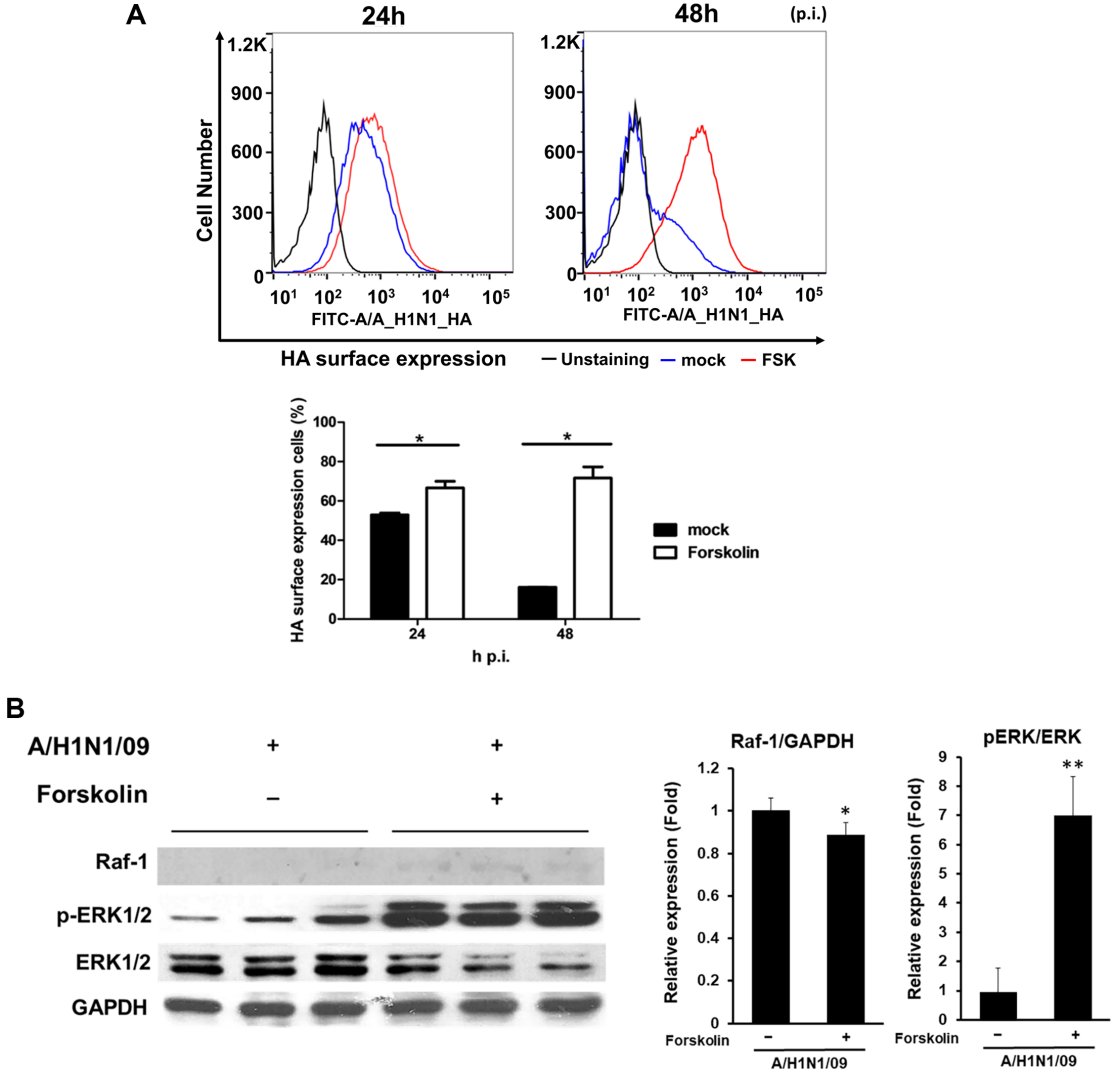
Expression of hemagglutinin (HA) on the cell surface and activation of ERK1/2 in the presence or absence of FSK. (**A**) Madin–Darby canine kidney (MDCK) cells were infected with 0.002 MOI of the A/H1N1/California/ 04/09 virus. The number of HA-expressing cells was measured using flow cytometry. In the histogram, the black line indicates unstained controls; a blue line indicates no treatment with FSK (mock), and the red line indicates treatment with FSK. The mean data are presented in the bar graphs. (**B**) Cells were infected with the virus under the same conditions as (**A**) and harvested 24 h p.i. for western blotting. The levels of Raf-1 are expressed as the ratio of the densitometric measurement of the corresponding internal standard (GAPDH) according to densitometric analysis. The level of pERK is expressed as the ratio of the phosphorylated proteins to the corresponding total proteins according to densitometric analysis. Data are shown as the mean (*n* = 3); *p < 0.05 and **p < 0.01.

Marjuki *et al*. [37] showed that membrane accumulation of the HA protein of the influenza A virus triggers nuclear export of the viral genome via PKCa and ERK signaling. In addition to examining ERK1/2 activity at different phases of the influenza replication cycle, we investigated the effect on ERK1/2 activity of HA expression on the cell membrane surface. HA expression was measured 24 h and 48 h after infection with A/H1N1/4/09. Infected cells were stained with an antibody against influenza virus A/H1N1 and analyzed using flow cytometry. HA surface expression was 0.25- and 4-fold higher in FSK-treated cells at 24 and 48 h p.i., respectively (Fig. 5A). At 48 h p.i., HA expression in untreated cells decreased compared to that at 24 h p.i., whereas HA expression in FSK-treated cells increased at 24 h p.i.. This result indicated that FSK-treated cells potentially generated more infectious viral particles at 48 h p.i. At 24 h p.i., the activated levels of Raf-1 and ERK1/2 in FSK-treated cells were higher than those in the untreated control cells (Fig. 5B). Based on these findings, we suggest that FSK contributes to influenza virus propagation by regulating ERK1/2 signaling.

## Discussion

In this study, we identified a new strategy to enhance the yield of cell-based influenza vaccines. Existing strategies for increasing influenza virus production are primarily based on identifying new cell lines with high susceptibility [8, 38] or modifying the host MDCK cell line [7, 9, 10, 39]. In contrast to these approaches, we focused on modifying the viral culture media to enable broader vaccine production applications. Initially, chemicals involved in cell cycle activation or inhibition (such as FSK, cilostamide, phytohemagglutinin, and FBS) were investigated for their cytotoxicity and potential to enhance viral titers. We found that FSK alone showed no cytotoxicity and effectively increased the titers of influenza virus (A/H1N1/California/4/09) in MDCK cells (data not shown). Based on these results, we analyzed the effects of FSK (a cAMP activator) on viral propagation. Several studies have shown that viral replication is regulated (enhanced or inhibited) by cAMP signaling. For example, the replication of hepatitis C and human immunodeficiency viruses is enhanced by modulating cellular levels of cAMP [40, 41]. In contrast, replication of vesicular stomatitis and herpes simplex viruses is inhibited by cAMP signaling [42]. To date, the link between influenza virus and cAMP-mediated modulation of viral replication is not understood. As mentioned above, FSK increases the production of influenza viruses in recent vaccine strains. Furthermore, FSK affected viral replication regardless of the treatment time before and after infection. Thus, FSK acts on MDCK cells but not on the viral life cycle. This finding was supported by the effect of FSK on increasing the production of influenza virus in sub-confluent but not in confluent conditions. Therefore, we conclude that FSK can be used to increase viral production in cell culture vaccine systems under appropriate conditions of cell confluency.

Notably, the association between cAMP and MDCK cell proliferation remains controversial [43]. Arginine vasopressin, a neurohypophyseal peptide hormone, prevents cell proliferation by increasing intracellular cAMP content [44]. In the present study, FSK-induced cAMP acted as a proliferation inhibitor in MDCK cells by inhibiting ERK1/2 activation via the PKA–Src axis. Thus, the G_0_/G_1_ to S transition was suppressed by the downregulation of cyclin D1 and CDK4 6 h after their release from synchronization; these were the main molecules regulating cell cycle progression in the G_0_/G_1_ phase [45, 46]. Notably, the influenza virus prefers the G_1_ phase of host cells for entry because of its sialic acid and lipid composition [31]. Therefore, we suggest that the hindrance of cell growth and cell cycle by FSK can enhance influenza virus infection. In contrast, FSK increased the activation of ERK1/2 during the late phase of influenza virus replication. ERK signaling activated by HA accumulation on the cell surface triggers the export of the viral genome and ribonucleoprotein (vRNP) [37]. FSK treatment increased HA expression on the cell surface and activated ERK1/2 signaling. We found that elevated cAMP levels increased HA expression on the cell surface; however, the underlying mechanism remains unclear. Notably, a previous study showed that increased RNA polymerase activity of the influenza virus enhances the activation of the HA-induced Raf/MEK/ERK signaling cascade [47]. Other studies have reported a link between cAMP and RNA polymerase in chickpeas [48, 49]. Therefore, we suggest that the link between cAMP levels and viral RNA polymerase activity in mammalian cells may explain the effects of FSK on influenza virus propagation. Further research is warranted to elucidate these mechanisms and assess the effects of FSK on viral RNA polymerase activity in mammalian cells.

**Fig. 6:**
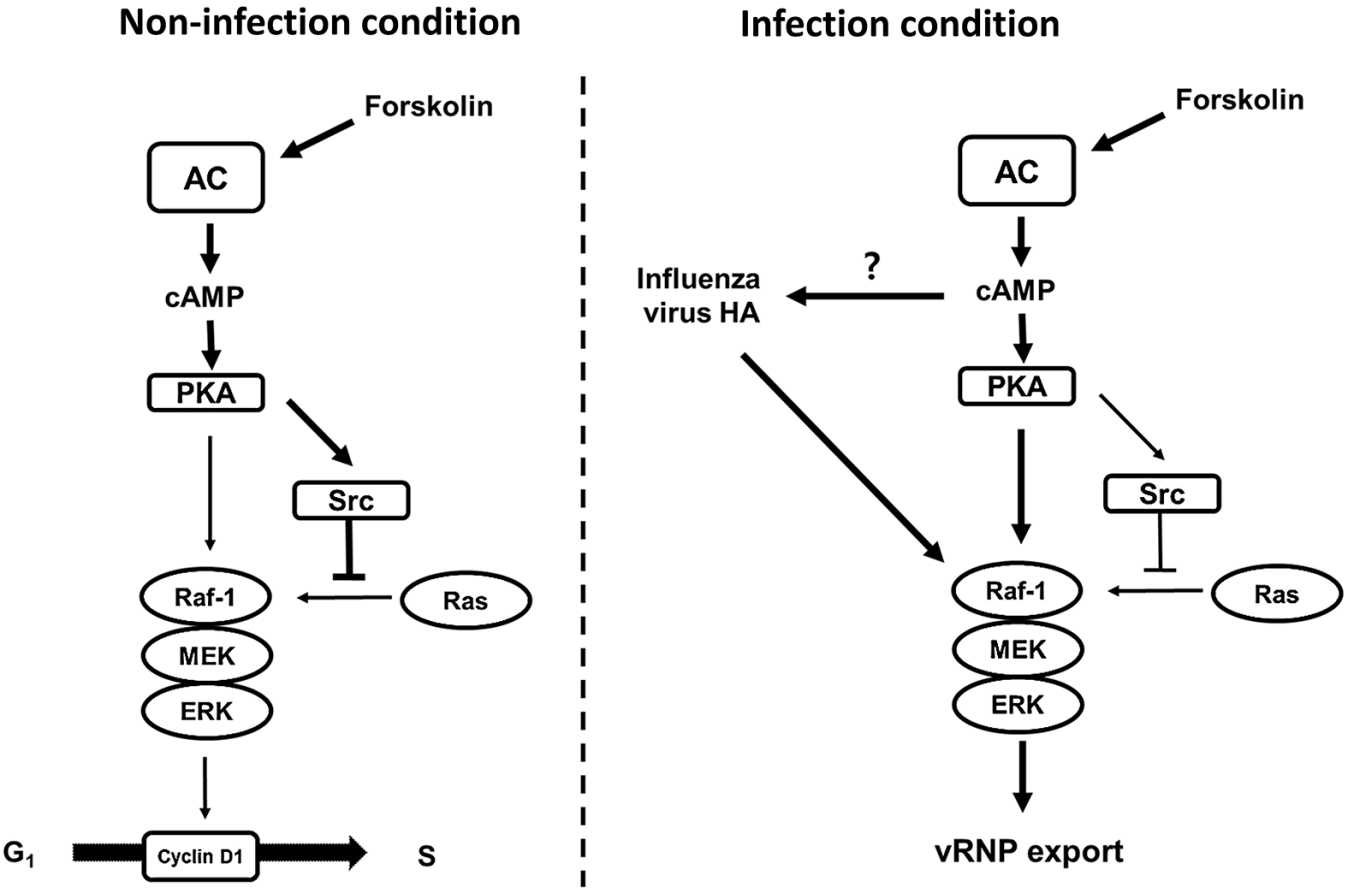
Postulated mechanism of action of FSK in MDCK cells under different conditions. (**A**) FSK inhibits cell proliferation by inhibiting the Ras-dependent Raf-1/MEK/ERK1/2 axis via PKA. (**B**) When MDCK cells are infected with the influenza virus, FSK synergistically activates ERK1/2 signaling and the viral HA protein accumulates on the cell surface.

In conclusion, we propose a mechanism for the role of AC in influenza virus growth (Fig. 6). Activated AC improves the environment for infection of MDCK cells with influenza virus. Increased amounts of sialic acid on the cell surface in the G_0_/G_1_ phase, which is delayed by FSK, may enhance viral infection in other cells. Moreover, in the late phase of infection, FSK treatment increased the intracellular levels of cAMP, which in turn induced increases in both the HA protein of the influenza virus on the cell surface and PKA activation; the synergistic activation of ERK1/2 serves then to increase vRNP export. Finally, FSK treatment assisted in the propagation of influenza virus. Therefore, this strategy may prove useful for the production of cell-based influenza vaccines by increasing yield and reducing the unit cost of production.

## Supplemental Materials

Supplementary data for this paper are available on-line only at http://jmb.or.kr.
