# Supplementary material for: The Adenylyl Cyclase Activator Forskolin Increases Influenza Virus Propagation in MDCK Cells by Regulating ERK1/2 Activity
Source: J Microbiol Biotechnol. 2023 Sep 4;33(12):1576–86. doi: 10.4014/jmb.2306.06027 (PMC10772552; doi:10.4014/jmb.2306.06027)
Supplement: Supplementary file 1 [file jmb-33-12-1576-supple.pdf]

Supplementary Figure. 1

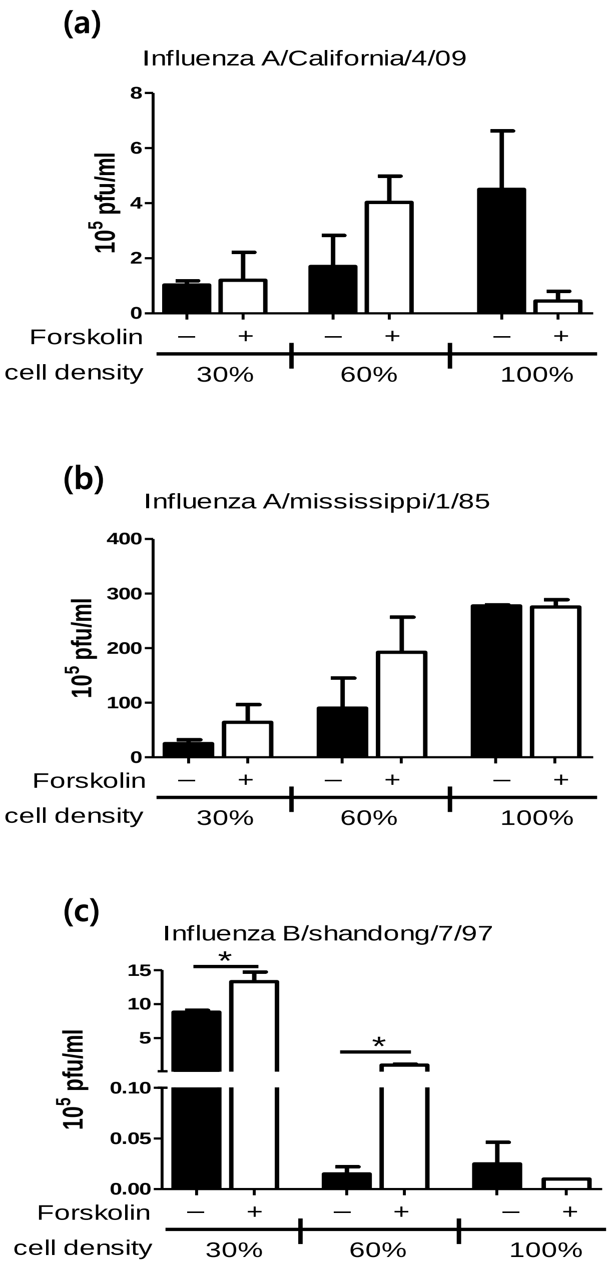

Supplementary table. 1

		G_0/G_1	S	G_2/M
2 hr	mock	20.67±1.55	42.73±2.06	23.33±2.10
	forskolin	19.37±1.48	40.87±3.16	25.53±2.97
6 hr	mock	31.67±3.49##	25.63±3.88##	23.97±5.48
	forskolin	20.00±1.31*	30.57±1.60#	35.63±1.19*#
12 hr	mock	26.83±2.67	27.40±3.96	26.93±1.50
	forskolin	39.17±2.29**###	22.03±1.53*##	18.97±1.81**###
24 hr	mock	32.27±3.59	23.03±1.70	27.37±2.41
	forskolin	34.37±1.50#	24.47±1.14	22.00±1.25*
